# Dietary inclusion effects of phytochemicals as growth promoters in animal production

**DOI:** 10.1186/s40781-017-0133-9

**Published:** 2017-04-17

**Authors:** Nidia Vanessa Valenzuela-Grijalva, Araceli Pinelli-Saavedra, Adriana Muhlia-Almazan, David Domínguez-Díaz, Humberto González-Ríos

**Affiliations:** 1 Laboratorio de Ciencia y Tecnología de la Carne, Centro de Investigación en Alimentación y Desarrollo A.C. (CIAD A.C.), Carretera a la Victoria Km. 0.6. C.P, Hermosillo, Sonora 83304 Mexico; 2 Laboratorio de Nutrición Animal, CIAD A.C, Carretera a la Victoria Km. 0.6. C.P, Hermosillo, Sonora 83304 Mexico; 3 Laboratorio Bioenergética y Genética Molecular, CIAD A.C, Carretera a la Victoria Km. 0.6. C.P, Hermosillo, Sonora 83304 Mexico; 4 Departamento de Nutrición Animal, Facultad de Zootecnia, Universidad Autónoma de Chihuahua, C. Escorza 900, Col. Centro, Chihuahua, Chihuahua 31100 Mexico

**Keywords:** Phytochemicals, Growth promoters, Meat production chain, Feed additives

## Abstract

Growth promoters have been widely used as a strategy to improve productivity, and great benefits have been observed throughout the meat production chain. However, the prohibition of growth promoters in several countries, as well as consumer rejection, has led industry and the academy to search for alternatives. For decades, the inclusion of phytochemicals in animal feed has been proposed as a replacement for traditional growth promoters. However, there are many concerns about the application of phytochemicals and their impact on the various links in the meat production chain (productive performance, carcass and meat quality). Therefore, the effects of these feed additives are reviewed in this article, along with their potential safety and consumer benefits, to understand the current state of their use. In summary, the replacement of traditional growth promoters in experiments with broilers yielded benefits in all aspects of the meat production chain, such as improvements in productive performance and carcass and meat quality. Although the effects in pigs have been similar to those observed in broilers, fewer studies have been carried out in pigs, and there is a need to define the types of phytochemicals to be used and the appropriate stages for adding such compounds. In regard to ruminant diets, few studies have been conducted, and their results have been inconclusive. Therefore, it is necessary to propose more in vivo studies to determine other strategies for phytochemical inclusion in the production phases and to select the appropriate types of compounds. It is also necessary to define the variables that will best elucidate the mechanism(s) of action that will enable the future replacement of synthetic growth promoters with phytochemical feed additives.

## Background

Growth promoters have been used in meat production for several decades to increase parameters such as daily weight gain and feed efficiency [1–3]. The positive effects of growth promoters meet the needs of the primary sector and impact the industrial sector and the end-users of meat production, such as consumers. In this context, there have been several benefits, including an increase in the yield of prime cuts [2, 3] and a decrease in the deposition of intramuscular fat, that result in the lean cuts that satisfy the demands of modern consumers [4–6].

On the other hand, an increasing number of consumers are concerned about the type and quality of their food. These consumers are rejecting the use of synthetic chemicals, such as those used in meat production, because their use is associated with human and animal health risks [3, 7]. In addition, producers are constantly warning of market failure due to the adoption of laws that prohibit the use of traditional growth promoters [8–10], such as antibiotic growth promoters (AGP) and the promoters used in the finishing stages. Due to these factors, the search for alternatives to synthetic growth promoters began several years ago, and various alternatives have been proposed that yield similar benefits.

There are a variety of growth promoters, mostly synthetic, that are being used in almost all stages of animal production. However, the search for alternatives is presently and has always been focused on a specific class, i.e., antibiotics [11], and it has ignored the compounds used in the finishing phase of animal production. In this context, secondary plant metabolites, i.e., phytochemicals, have emerged as alternative growth promoters. Their use was first proposed a few decades ago, but their effects on performance have been inconsistent [11]. However, most research has focused on analyzing the effects of phytochemicals on the productive behavior of animals as a supplement, neglecting the benefits of traditional growth promoters throughout the entire meat production chain. Therefore, it is necessary to understand the effects of phytochemical feed additives in each sector of the meat production chain to develop strategies for their optimum use for the benefit of all members.

This review summarizes the scientific knowledge on the use of dietary phytochemicals as animal growth promoters in cattle, pig and poultry production, as well as current strategies for their inclusion in animal production systems (amounts and period of inclusion). An overview of recent knowledge of the effectiveness and possible modes of action of phytochemicals along the meat production chain are provided.

## Phytochemicals as an alternative to animal growth promoters: current situation

Due to restrictions in several countries on the use of antibiotic growth promoters in meat production (poultry, beef and pork), replacing these compounds is of great interest [9, 12]. Thus, various alternatives, including dietary supplementation with secondary plant metabolites, referred to as phytochemical feed additives, phytogenic feed additives, phytobiotics, or herbal and botanical compounds [13–18], have been proposed. Initially, researchers began experimenting with the addition of herbal extracts, such as essential oils, and they are now experimenting with the addition of isolated compounds. The use of phytochemicals in animal feed is accepted by consumers as herbal medicines have been consumed by humans for centuries [12].

Currently, phytochemicals have not only been proposed as a replacement for antibiotic growth promoters but also for other anabolic compounds that are used to increase animal growth [19–21]. These compounds are being replaced because current trends indicate that consumers are increasingly rejecting the use of synthetic substances in food production; therefore, plant-derived compounds with growth-promoting activity, also known as phytogenic compounds, are gaining a presence in the feed additive market [11, 22].

## Types of phytochemicals currently used as growth promoters

Phytochemical feed additives are an extremely large group of compounds with great diversity in chemical structure and bioactivity [12, 23]. The active compounds in plants vary widely depending on intrinsic factors, such as the plant part used, the harvest season and the geographical origin, and extrinsic factors, such as the additive production technique [13]. The diversity of secondary plant derivatives is the result of an evolutionary process through which plants have acquired enhanced defenses against attacks by microorganisms, insects and other animals [24]. Phytochemicals present several biological properties that have made them attractive for use as growth promoters in animal production, including antimicrobial, antioxidant, anti-stress, and nutrigenomic effects on the development of immunity [22, 25, 26]. Therefore, these compounds are a significant source of a variety of compounds with different biological activities that have the potential to promote the growth of producing animals [22].

**Fig. 1:**
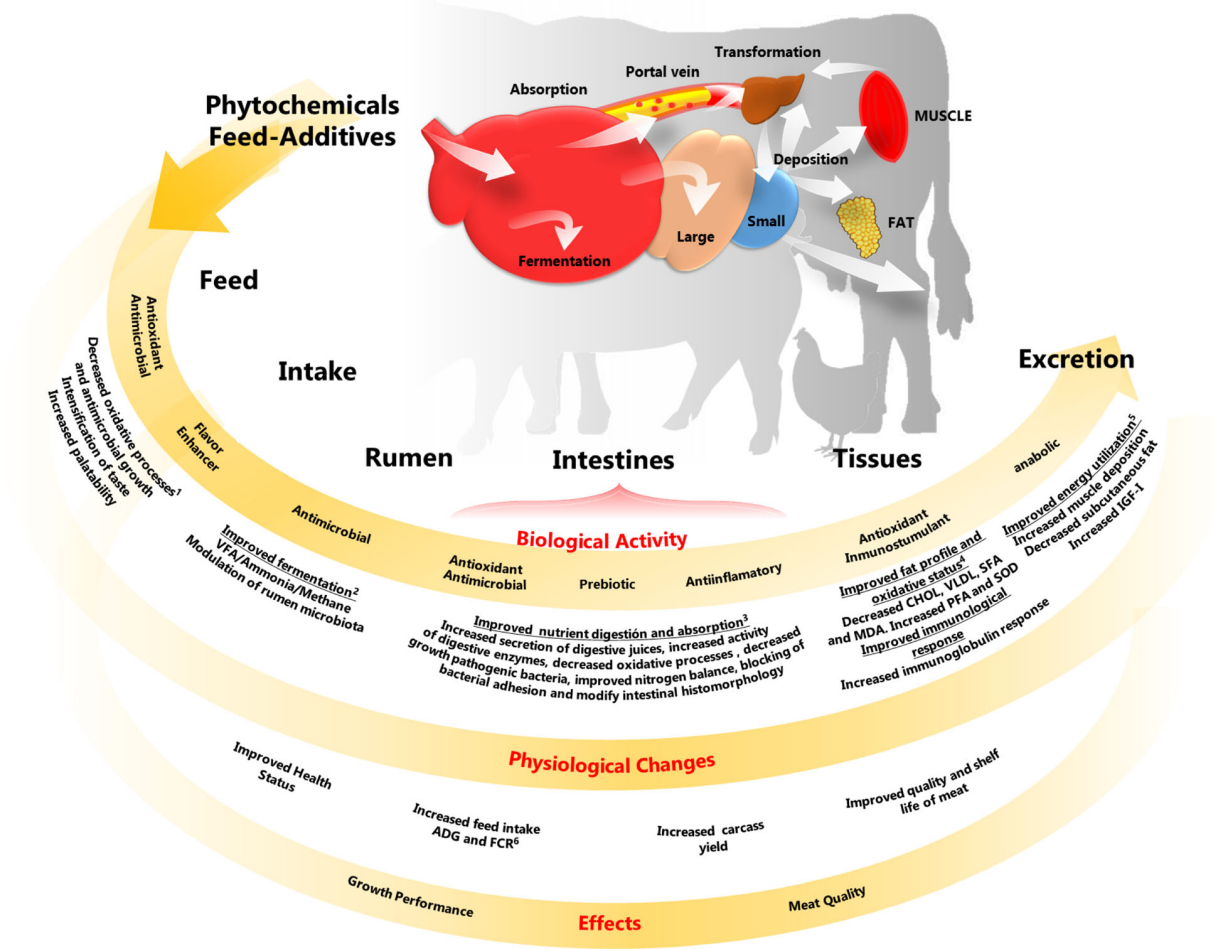
Schematic description of main mechanism of action and effects of dietary phytochemicals feed- additives

**Fig. 2:**
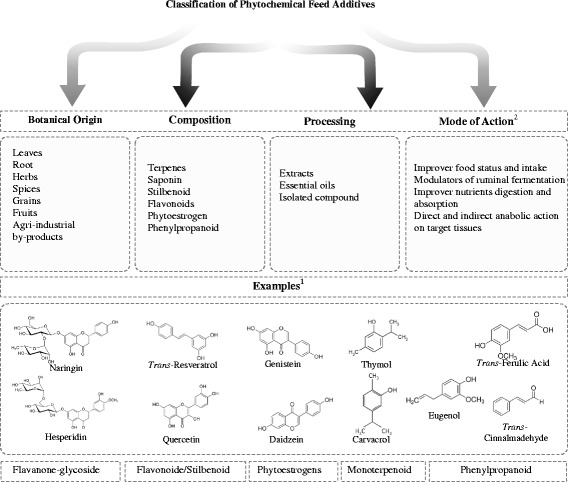
Classification proposed and several examples of phytochemicals used as growth promoters additives

A wide variety of phytochemical feed additives have been used experimentally in animal production; it is difficult to classify them, in part, because there are no concise definitions [13–15]. A confusion source is whether to define the entire plant as a phytochemical feed additive. Several authors have proposed various classifications based on botanical origin, composition and processing. Another possible method is based on the proposed mechanism of action (Fig. 1), but some phytochemicals, due to their wide range of biological activities, may exert their effect through different mechanisms, thus complicating their classification. Moreover, most studies have focused on the use of these compounds to replace the growth promoters used in the early stages of the animal production, ignoring those that are employed in the finishing phases (mainly in pigs and cattle); some examples are suggested in Fig. 2.

Initial investigations suggested the use of herbal and spice extracts, called essential oils, as phytochemical feed additives [27]. The use of these compounds has begun, including some compounds that are rich in lipids, such as carvacrol and thymol (monoterpenes). Furthermore, the use of agro-industrial by-products to isolate phytochemical growth promoters has been proposed to reduce the environmental impact; an example is the ferulic acid, which is isolated from nejayote [28].

## Proposed mode of action of phytochemical growth promoters

The action mechanisms underlying the use of phytochemical feed additives as animal growth promoters have not been completely elucidated yet, even though these chemicals have been used for a long time as medicines, flavors and food preservatives [18]. Based on the different biological activities of phytochemicals, four principal mechanisms have been proposed (Fig. 1) that support the physiological changes observed in the studied animals and explain the effects on growth performance, carcass characteristics and meat quality: 1) Improver of food status and animal feed intake; 2) Modulator of ruminal fermentation; 3) Improver of nutrient digestion and absorption; and 4) Source of direct and indirect anabolic activity on target tissues [12, 22, 23, 25, 26]. The proposed mechanism of action for a particular phytochemical feed additive depends largely on its structure, dosage, and pharmacokinetics, as well as the animal species, productive stage and administration period. On the other hand, the observed growth promoting effect may be due to several mechanisms, as a result of the various biological activities of the phytochemicals. However, information on what determines various mechanisms within the same experiment has not been reported; this may be due to a lack of studies on the mechanisms of a specific phytochemical in a given species.

### Improver of food status and animal feed intake

Some phytochemical additives enhance the flavor and palatability of feed, which improves feed intake and productive performance [29–33]. This result may be related to several biological activities, such as antioxidant, antimicrobial and flavor enhancer effects [34–36]. The proposed modes of actions are the following: 1) Improved the feed antioxidant status 2) Decreased the antimicrobial colonization, or 3) Increased the stimulation of appetite. Some phytochemicals can excite the olfactory nerves and taste buds [37, 38]. All of these effects can cause positive results like higher feed consumption and weight gain [39–41].

Janz et al. [31] found that pigs preferred the feed that was supplemented with garlic or rosemary over the feed that was supplemented with oregano or ginger. Franz et al. [42], based on 23 studies with broilers, mentioned that the feed conversion ratio was improved with phytochemical additives. Furthermore, several studies have reported a decrease in feed intake as a result of the high inclusion level (>1500 mg/kg) of phytochemical feed additives and the intrinsic properties of some compounds, such as a strong odor and flavor [43, 44]. Thus, the high levels of phytochemical additives used in animal feed must be avoided, especially for porcine species, because of their sensitive palates [45]. However, studies testing these characteristics are limited, and the assumption that phytochemical additives improve feed palatability has not been completely justified yet.

### Modulators of ruminal fermentation

Microbiota in the rumen and ruminant physiology are manipulated in order to enhance the productivity and health status of an animal. The activities of the rumen microbiota, including protein digestion and synthesis, carbohydrate digestion and vitamin synthesis, are important for obtaining an adequate production profile of volatile fatty acids (propionate, acetate and butyrate), mainly propionate, since most energy maintenance and growth performance is linked to it [46, 47]. In this sense, as previously mentioned, a property of phytochemicals (mainly tested in essential oils) that has been known for centuries is their antimicrobial capacity [24–48]. Based on this biological activity, several researchers have proposed the use of these compounds as a replacement for antibiotic growth-promoting modulators of the rumen microbial population [49, 50].

There are four recognized modes of action that explain how phytochemical feed additives exert their antibacterial effect and the changes that occur in rumen microbiota: 1) inhibition of cell wall synthesis, 2) disruption of cell wall structure (altering the permeability of the cytoplasmic membrane), 3) inhibition of nucleic acid synthesis, 4) inhibition of protein synthesis and inhibition of a unique bacterial metabolic pathway. These actions lead to the collapse of core cellular activities and, consequently, result in bacterial death [51]. Changes in the rumen microbial population due to the use of antibiotic growth promoters may result from changes in rumen fermentation, which reduces methane production and increases the number of volatile compounds, especially fatty acids such as propionic acid. Overall, these changes improve feed efficiency and average daily gain [27, 52].

In most cases, the positive effects of essential oils, such as an increase in propionate and decreases in acetate, methane and the production of ammonia nitrogen, have been reported without decreasing the total production of volatile fatty acids [53]. Furthermore, some studies showed beneficial changes in rumen fermentation and found a decreased acetate/ propionate ratio [52, 54–56]. Dong et al. [57] evaluated the effects of adding lucerne extract, *Artemisiae annuae* extract and a mixed herbal medicine to different goat diets on in vitro fermentation and methane production. They observed that these phytochemicals reduced methane production, increased the propionate concentration and decreased protozoa numbers. Studies suggested that several phytochemicals prevent protein degradation as a result of a decrease in the ammonia nitrogen concentration [53]. However, even when phytochemicals have an effect on the modulation of rumen microbiota, their effect on animal performance remains unclear; this may be partly explained by the fact that most research has been conducted in vitro with isolated cultures, and there are no in vivo studies to confirm if this effect on microbiota has consequences on performance [49, 58].

### Improver of nutrient digestion and absorption

Phytochemicals exhibit various biological activities that are related to the functions of the intestinal tract, such as digestive secretions and nutrient absorption [11]. These biological activities are primarily attributed to: 1) increased digestive secretions (saliva, digestive enzymes, bile, and mucus) [59, 60]; 2) decreased bacterial counts and pathogen loads via an antibacterial effect in the intestinal lumen [61]; 3) developed antioxidant and anti-inflammatory activities in the intestinal lumen, resulting in improved gut morphology [37, 62, 63]; 4) prebiotic activity [64], and 5) reduced fermentation products, such as nitrogen compound waste [65]. These effects may improve intestinal health and the absorption and utilization of nutrients, thus improving the growth of the animals within their genetic potential [18, 22]. The actions that improve intestinal tract function are very complex, thus several mechanisms might be involved.

Accordingly, an increase in digestive secretions is one of the most important effects in improving nutritional status [66]. The actions that can produce these effects are: 1) increased salivary gland secretion (increased amylase), stimulation of mucus secretion in the stomach and intestine (which prevents the adhesion of pathogens on the intestinal villi and promotes the development of intestinal villi) and increased gastric secretion (activation of pepsin) [60, 66–69]; 2) increased secretion and function of pancreatic and intestinal enzymes, such as lipases, trypsin, chymotrypsin, carboxypeptidase and lipase (which accelerate digestion and shorten the time of feed passage through the digestive tract), synthesis of bile acids in the liver and their excretion in bile (improved digestion and absorption of lipids) and increased activity of digestive enzymes in the gastric mucosa [59, 70, 71].

Phytochemical feed additives, such as capsaicin, have been shown to be effective in stimulating salivation [67]. Jamroz et al. [68] concluded that the increased mucus secretion in the glandular stomach and jejunum wall of broilers that were fed a mixture of carvacrol, cinnamaldehyde and capsaicin could be responsible for the reduction of adhesion pathogens. Manzanilla et al. [70] and Jang et al. [71] found a stimulating effect on the pancreatic enzymes (trypsin, amylase and maltase activity) of pigs and broilers that were fed cinnamaldehyde and an essential oil blend (thymol, eugenol and piperine), respectively. The increased activity of the digestive enzymes promoted an increase in the gastric retention time of the ingested feed, thus resulting in improvement of its digestibility and the availability of nutrients [59, 72].

As indicated by the foregoing discussion, modulating the gut microflora and controlling the adhesion of antimicrobial pathogens can maintain intestinal epithelium integrity and health status by reducing toxins and increasing the availability of nutrients for absorption [11, 59]. Due to their hydrophobicity, most of the phytochemical feed additives can interact with the cell membrane, increasing membrane permeability and resulting in ATP disruption, the loss of cell content, and cell death. Accordingly, some in vivo studies with pigs found that dietary supplementation with a commercial mixture of phytochemicals showed antimicrobial activity; they found a decrease in fecal *Salmonella* and *E. coli* counts [61], an increase in *Lactobacillus spp.* counts and a decrease in diarrhea scores when benzoic acid and thymol were used [63]. However, in vivo studies that evaluate these parameters are limited, so it is difficult to form conclusions about the antimicrobial efficacy of phytochemical feed additives.

Additionally, reactive oxygen radicals produced during the digestion process can attack the surface of the intestinal mucosa, preventing the absorption of nutrients. Antioxidant activity is responsible for decreasing these reactive oxygen radical products by neutralizing them and thus maintaining better conditions at the intestinal surface. Placha et al. [73] found that broilers fed with thymol had lower malondialdehyde (MDA) content in the duodenal mucosa and better intestinal integrity in broilers fed with thymol.

The anti-inflammatory activity of phytochemicals is another property of great interest because it suppresses the metabolism of inflammatory prostaglandins. Terpenoids and flavonoids are some phytochemicals that have been reported to possess anti-inflammatory activity [37]. In addition, Rubio et al. [74] propose that phytochemical feed additives may act by over-expressing antioxidant enzymes, which might down-regulate the inflammatory process. Antioxidant and anti-inflammatory activities in the intestinal lumen resulted in improved gut morphology [75]. Another activity that has been reported for phytochemical feed additives is that they can act as prebiotics; in other words, they can indirectly improve the growth of intestinal flora, such as *lactobacilli* and bifidobacteria, that use these compounds in their own metabolism [63, 64, 76].

Positive effects on the intestinal morphology have been shown in broilers and pigs. Cardoso et al. [40] found that the dietary supplementation of broilers with 60 mg/kg of piperine increased the absorption surface of the duodenum and the ileum, but higher doses (120 and 180 mg/kg of piperine) caused negative effects, because they decreased absorption surfaces. In the same species, Kanduri et al. [41], Khalaji et al. [62] and Amad et al. [77] reported that a blend of essentials oils, a blend of phytochemicals and a blend of thymol, anethole and cinnamaldehyde, showed the best results in the intestinal micrometry, such as improved villus/crypt ratios. In pigs, Diao et al. [63] reported improvements in intestinal lumen morphology. Intestinal ammonia is considered an important health stress. Added to that, Bartoš et al. [30] found that supplementation with a commercial mixture of essential oils in pigs reduced the ammonia and methane emissions per animal per day; this result is possibly due to the inhibition of the activity of the microbial urease enzyme. Another phytochemical feed additive that has been proposed to decrease the production of ammonia in vitro is saponins, through its mechanisms are not yet known [78]. All these observations support the hypothesis that phytochemical feed additives may favorably affect gut function; however, the number of in vivo studies with swine and poultry is still quite limited.

### Direct and indirect anabolic activity on target tissues

Recently, some phytochemicals have been proposed to exert direct and indirect effects on animal metabolism, similar to those observed with the use of anabolics; thus, they may act as growth promoters by modulating animal metabolism in favor of increasing muscle tissue. This effect is the reason that phytochemicals like genistein, daidzein, soybean isoflavone and ferulic acid are used in the finishing phase of production animals. However, several phytochemicals have been tested in early stages and have been shown to have an anabolic activity [19–21, 32, 79]. In addition, some studies have proposed two possible mechanisms of action for these results. On the one hand, the phytochemicals can act by modulating animal metabolism in a way that is similar to the action of β-adrenergic agonist compounds. This mechanism was proposed based on the structural similarities between some compounds of plant origin (e.g., hydroxycinnamic acid derivatives of the amino acid phenylalanine) and catecholamines, which are natural animal hormones [19, 80, 81]. These hormones interact with β-adrenergic receptor agonists to change animal metabolism, mainly by increasing protein synthesis and lipolysis and by decreasing lipogenesis [82]. Additionally, porcine and bovine studies showed that an anabolic effect is possibly produced by some phytochemical feed additives, such as a blend of plant extracts [21, 83] and ferulic acid [80], as reflected by increased serum IGF-I levels. On the other hand, the second mechanism is closely related to catecholamines and, more specifically, to norepinephrine. Accordingly, some studies noted that the increased plasma norepinephrine levels in humans and animals treated with phytochemicals are the result of the inhibition of catechol-*o*-methyltransferase; this result occurs due to the similar structures of some phytochemicals (or their metabolites) and normetanephrine, a norepinephrine metabolite [84–86].

Other biological activities that are associated with phytochemical additives are immunostimulatory and antioxidant effects; thus, these effects can result in favorable conditions for animal health, and they can focus on target tissues, such as muscle and the intestinal lumen, by promoting their growth and improving their antioxidant status. Several in vivo studies have reported an antioxidant action in meat as a result of supplementing animal diets with phytochemical feed additives, and the use of these additives has shown positive effects against lipid oxidation. In addition, some studies attribute these effects to the phytochemicals that can incorporate into the phospholipids of the plasmatic membraneand interact with the antioxidant system. For example, vitamin E [87, 88] enhances the antioxidant activity system, therefore contributing to a longer meat shelf life [87, 88]. Additionally, several studies have found an increase in the activity of some antioxidant enzymes in muscle and serum [32, 87, 89]. For instance, in studies by Jiang et al. [32] and Kamboh and Zhu [89], supplementation to broilers with soybean isoflavone and with genistein and hesperidin, respectively, resulted in decreases in malondialdehyde (MDA) content, antioxidant capacity, and superoxide dismutase, and improvements in catalase activity in serum and breast muscle. In pigs, Li et al. [87] reported similar results when ferulic acid was added in the diet (decreased MDA and increased hepatic glutathione peroxidase activity). Additionally, some studies in broilers [29, 89, 90] found that supplementation with thyme and cinnamon essential oil, an essential oil blend (oregano, anise and citrus peel), genistein and hesperidin decreased the cholesterol, VLDL and triglyceride contents in serum and skeletal muscle.

On the other hand, plants contain compounds that can act as an immunological stimulator; however, this activity is not fully known yet. The phytochemicals that have been shown to possess immune-stimulatory properties are flavonoids, vitamin C and carotenoids [90]. Phytochemical feed additives may help the immune response when animals are in immune-suppressed conditions by improving the activity of lymphocytes, the immunoglobulin response, monocytes, macrophages and NK cells and, consequently, increasing phagocytosis and stimulating interferon synthesis [39, 62, 91–93]. The immune-stimulant activity may improve duodenal function and increase the availability of nutrients for absorption; consequently, this activity could result in an improvement of the general status of the animal by transforming these nutrients in energy sources and tissues and by enhancing its genetic potential for growth [94]. In this sense, Alipour et al. [39] reported an increase in the immunological response of broiler serum after supplementing with an essential oil of thyme.

## Dietary inclusion of phytochemicals and their impact on the meat production chain

For several years, growth promoters have been widely used by animal producers in the meat production chain to not only improve growth performance but also improve carcass quality characteristics and maintain meat quality characteristics, although the effects on carcass and meat quality remain a topic of discussion [1, 3]. However, changes in the legislation of several countries and consumer preferences have led to the search for new, alternative growth promoters, such as the use of phytochemicals in animal feed [18, 22, 95]. While this alternative should produce similar or better effects than traditional growth promoters, most of the studies and conclusions on the effects of phytochemical feed additives are based on human medicine. In addition, many of the studies have proposed the use of these supplements as alternatives to antibiotic growth promoters (which are mainly used in the initiation and development stages of production), but very few have evaluated replacement of the growth promoters that are used in the finalization phase (e.g., anabolics). There have also been few in vivo studies (mainly limited to poultry and swine) or performance tests to assess the changes in production parameters, i.e., carcass and meat quality, to support the addition of phytochemicals as growth promoters in animal production.

## Growth performance

The growth-promoting modulators of ruminal fermentation and the modulators of the intestinal system and animal metabolism that are currently used in animal production have been shown to have positive effects on growth performance, with increases in daily gain weight and feed efficiency between 10 and 20% [1, 2, 96].

### Effects on poultry growth

A literature review indicated that most of the in vivo studies evaluating the effect of adding phytochemicals to broiler diets have shown positive effects on growth performance parameters, such as weight gain, feed intake, and feed conversion (Table 1); these results are similar to or even better than those observed in treatments with a positive control (e.g., tylosin). In these studies, most of the phytochemicals (plants extracts: derived from thyme, oregano, agronomy, clove, lemon, balm, red pepper, black cumin seed, Artemisia leaves, *Macleaya cordata*; essential oils: thyme, cinnamon, oregano, anise, citrus peel and rosemary) were added in each of the three main production phases, and primarily commercial extracts of plants (leaves) and herbs were used as feed additives. Some studies evaluated the addition of simple phytochemicals, such as isoflavone, quercetin, naringin, hesperidin and piperine [32, 97–99].

**Table 1:** Effects of phytochemicals feed-additives on productive performance, carcass and meat quality of broiler

Reference	Phase^a^	Phytochemicals	Dosage	Productive performance ^b^	Carcass quality^c^	Meat quality^d^
[29]	G and F	Thyme and cinnamon	100 and 200 ppm	↑ BWG, FI and FCR	200 ppm ↓abdominal fat %	-
[32]	G and F	Soybean isoflavone	10 to 80 mg/Kg	10 and 20 mg/Kg ↑ BWG and FI	-	40 mg/ kg ↑ WHC and pH 40 or 80 mg/Kg ↑lightness ↓MDA
[39]	I-F	Thyme extract	50, 100, 200, or 400 ppm	↑ BWG	-	-
[40]	I-F	Piperine	0, 60, 120, and 180 mg/kg	In the final period: 60 mg kg 1 ↑ WG and FCR	-	-
[41]	I-F	AV/AGP/10 essential oils (*Allium sativum, Zingiber officinale, Trigonella foenum graecum, Eruca sativa* & many others in a fixed concentration)	250 and 500 g/ton of feed	Herbal ≈ antibiotic	The AV/AGP/10 ↑ CY and dressing percentage	-
[62]	I and G	Black cumin seed, A*rtemisia* leaves, *Camellia* l.	0.3 and 0.5 g/kg	*Artemisia* ↓FI up to 21 d of age. -Black cumin ↑BW at 21 and 42 d of age and ↓FCR. *Camellia* ↓BW, FI, FCR	NE	-
[89]	I-F	Genistein (G) hesperidin (H)	5 mg/kg G H5, 10 20 mg/kg of mixture	-	-	In breast muscles: ↑PFA, n-6/n-3 and PFA/SFA
[90]	G and F	Oregano, anis and citrus peel	125 ppm	↑ FCR ≈ antibiotic	NE	↑tenderness ↑acceptability
[97]	I-F	Quercetin	0.5 and 1 g/kg of feed	Poorer FCR	-	↑ MDA
[98]	I-F	Naringin and Hesperidin	0.75 and 1.5 g/kg	NE	NE	↑ Oxidative stability
[99]	I-F	*Moringa Olifeira* leaf meal	1, 3 and 5 g : 3, 9 and 15 g: 5, 15 and 25 g per kg of feed	↑ FCR ↑BW and ADG	-	-
[100]	I-F	Oregano and vitamin E	100 mg/kg of feed	NE FI, BWG, and FCR		-The MDA in oregano was the second highest, at 9 d of storage
[101]	G	Oregano, clove, cinnamon, red pepper	100 ppm	NE	-	-
[102]	I and G	Sangrovit® *Macleaya cordata* extract	20 mg/Kf of feed 0.24 mg/Kg of sanguinarine	NE FI, BWG, FCR	NE	-
[103]	I	Rosemary and oregano Commercial blend of essential oils	50 and 100 mg 1 g of comercial	BWG and FCR ≈ avilamycin	-	-
[121]	I-F	Biostrong 505 Biostrong 510	0.05%	↑ Growth	Biostrong 505 ↑ CW and BR	-
[104]	F	Clove powder Agrinomy extract Lemon balm	1% clove / 0.2% lemon balm extract or agrynomy extract	↑ BWG - first period ↓FCR	↑ CW	↑ Crude protein ↓ Fat ↑ sensory evaluation
[106]	I-F	oregano, anis and citrus	125 g/t	↑ BWG	-	-

^a^Feeding phase: I, initial; G, growing; F, finalization

^b^ *BW* body weight, *BWG* body weight gain, *FCR* Feed conversion ratio, *CFI* cumulative feed intake, *FI* feed intake, *NE* no effect

^c^ *CY* Carcass yield, *CW* carcass weight, *BR* breast weight

^d^ *FA* fatty acid, *MDA* malondialdehyde, n-6/n-3 fatty acid ratio, *PFA* polyunsaturated, *SFA* saturated fatty acid, *WHC* water holding capacity

Only a few studies have reported no significant effects [97, 100–103] or negative effects. For example, in the studies conducted by Goliomytis et al. [98] and Marcinčák et al. [104], who supplemented animal diets with quercetin and extracts of *Camellia* leaves, respectively, a decrease in productive performance, in terms of final body weight, feed intake and feed conversion, was observed.

Positive results on productive parameters were mostly observed by adding phytochemical mixtures throughout all of the production phases, while null or adverse effects appeared when phytochemicals were supplemented in a single phase and/or as isolated compounds (e.g., quercetin, genistein, isoflavone) [32, 89, 105, 106].

### Effects on pigs

In contrast to the broiler studies, few studies have evaluated the addition of phytochemical feed additives throughout all of the production phases of pig; most have only evaluated supplementation in the initial or finishing phases of intensive feeding (Table 2). The phytochemicals used in this species are varied, but they predominately consist of plant extracts and herbs and, less frequently, isolated compounds, such as ferulic acid, resveratrol and phytoestrogens (genistein and daidzein). In this sense, positive effects on daily gain, feed intake and feed conversion have been observed [19, 21, 30, 61, 63, 83, 107–109]. Zhou et al. [110], Biquan et al. [111], Janz et al. [31] and Rossi et al. [112] found no significant differences in any productive parameter, while that Bruno et al. [113] found positive results by using tylosin and a mixture of plant extracts during the finishing phase. The phytochemical feed additives that have been used experimentally in pigs have mainly been proposed as alternatives to antibiotic growth promoters. Although most of the porcine studies have used phytochemicals in the finishing phase, only an in vivo study by Herrera et al. [19] proposed ferulic acid as an alternative to type β-adrenergic agonists as animal metabolism modulators. This study found similar effects on productive parameters as those observed in animals treated with ractopamine hydrochloride.

**Table 2:** Effects of phytochemicals feed-additives on productive performance, carcass and meat quality of porcine

Reference	Phase^a^	Phytochemicals	Dosage	Productive performance^b^	Carcass quality	Meat quality^c^
[19]	F	Ferulic acid FA	12 and 15 ppm	FA ≈ ractopamine	average fat ≈ ractopamine	-
[21]	G	*Phlomis* *umbrosa Turcz*, *Cynanchum wilfordii Hemsley*, *Zingiber* *officinale Rosc*, and *Platycodi Radix*.	0.05 and 0.15%	↑ ADG NE in ADFI and FCR	NE	
[30]	G and F	Essential oil blend Dried herbs, spices and *Quillaja saponaria* saponins		↑ ADG, FI and BW	-	-
[31]	G	Essential oils and Oleoresins (rosemary, garlic, oregano and ginger)	0.05%	↑ FI	NE	Minimal impact on the lipid oxidation
[33]	I-F	Tangerine	8%	-	-	NE
[44]	G	Buckwheat, thyme, curcuma, black pepper and ginger	-	↑ Improved ADG	-	-
[61]	I	Respig®; containing resveratrol and Biomin® PEP; containing essential oil blend	0.2% resveratrol and 0.0125% EO	↑ FCR	-	-
[63]	I and G	Benzoic acid and Thymol	100 or 200 mg of Thymol/Kg	200 mg of thymol ↑FCR	-	-
[83]	F	Biosun® Herbal extract *(Astragalus membranaceus Bunge, ycium barbarum L., Atractylodes macrocephala Koidz, Shenqu*, and *Glycyrrhiza uralensis Fiseh)*	250 mg /kg diet	↑ ADG	-	-
[87]	F	Ferulic Acid (FA) Vitamin E (VE) Individually or in Combination	0 or 100 mg/kg	-	↑ pH_45_min value	The combined addition of FA/ VE showed negative synergistic effects in inhibiting MDA production
[107]	G	Soy genistein	0, 200, 400, and 800 ppm	↑ growth performance	-	-
[108]	G	Daidzein	0, 200, 400, or 800 ppm	↑ ADG and FCR during periods of peak viremia	-	-
[110]	G and F	*Captis chinensis* herb extract		NE	-	↑ Meat color, pH, WHC and UFA ↓SFA
[111]	F	Phytochemical additive blend	0.04%	NE	-	↓MDA content and ↑ SOD activity
[112]	I-F	Verbenaceae (*Lippia* spp.) leaves	5 mg/kg feed	NE	NE	↓TBARS values in the raw meat
[113]	I-F	*Rosmarinus officinalis, Mentha piperita, Lippia sidoides and Porophyllum ruderale*	2000 ppm	NE	-	NE
[155]	F	Oregano	1000, 2000 or 3000 ppm	-	-	1000 ppm ↓Lipid oxidation

^a^Feeding phase: *I* initial, *G* growing, *F* finalization

^b^ *ADG* average daily gain, *BW* body weight, *ADFI* average daily feed intake, *DMI* dry matter intake, *FCR* feed conversion ratio, *FI* feed intake, *NE* no effect

^c^ *FAP* fatty acid profile, *MDA* malondialdehyde, *SOD* super oxide dismutase, *SFA* saturated fatty acid, *TBARS* thiobarbituric acid reactive substance, *UFA* unsaturated fatty acid

### Effects on bovines and ovines

Research involving ruminants has mainly been focused on inducing changes in the microbial populations of the rumen and its subsequent effects on ruminal fermentation; in vitro studies have been conducted with the goal of improving energy production and metabolites to increase muscle tissue [53, 114] (Table 3). However, contrary to reports involving poultry and pigs, there are few in vivo studies that evaluate the effects of dietary supplementation with phytochemicals on beef cattle and ovines. Most studies in cattle have evaluated the effects of the addition of essential oils in the growth and finishing phases; eugenol, hydroxycinnamic acids, cinnamaldehyde and ferulic acid are the simple phytochemicals that have been evaluated in these species. In contrast to the effects observed on productive parameters in poultry and swine, these parameters were not modified or were only minimally modified in bovines [27, 55, 115, 116]. Yang et al. [33] reported negative effects resulting from a high inclusion level (1600 mg/d) of eugenol during the finishing phase. Lin et al. [56] conducted two experiments with different doses (0 to 500 mg /l) of eugenol, carvacrol, citral and cinnamaldehyde and observed a decrease in methane production, protozoa, fungi, *Ruminococcus fibrisolvens* and *Fibrobacter succinogenes* at the higher dose. However, high phytochemical concentrations inhibited the growth of microorganisms and ruminal fermentation, which are important activities for the conversion of nutrients in muscle tissue [118, 119].

**Table 3:** Effects of phytochemicals feed-additives on productive performance, carcass and meat quality of bovine and ovine

Reference	Phase^a^	Phytochemicals	Dosage	Productive performance ^b^	Carcass quality	Meat quality^c^
[20] Bovine	F	Ferulic acid FA		↑ Feedlot performance	FA ↑ carcass characteristics and wholesale cut yield	FA (30 days) ↑ Tender, juicier and flavored meat smaller increases in TBAs values
[33] Bovine	F	Cinnamaldehyde CIN	-	↑ FI (initial month) Minimal effects on ADG and FE	Minimal effects on carcass traits similar to positive control	-
[52] Bovine	F	Alfalfa extract, anise, capsicum Mixture of Cinnamaldehyde and eugenol (CIE)	-	CIE and alfalfa ↓DMI and water intake	-	-
[55] Bovine	F	Thyme and cinnamon essential oils	5 g/d/calf	NE	-	-
[79] Ovine	F	Ferulic acid	300 mg of FA/animal	-BWG and ADG tended to ↓ d 17 to 34	NE	-
[109] Ovine	F	Hesperidin	1500 and 3000 mg/kg	NE	NE	↓ Lipid oxidation values
[115] Bovine	G	*Stryphnodendron adstringens,* Commercial product 1 (essential oils) and commercial product 2 (cashew nut)	15 g∙steer − 1∙d − 1	≈ monensin	NE on fat thickness	-
Ovine	G	Carvacrol	0.30 and 0.35 g/Kg of Dry matter	NE	NE	-
[116] Bovine	G	CIN and Thymol blend	100 or 200 mg/Kg of diet	Minimal effect on growth rate and FE	-	-
[122] Ovine	F	Cinnamaldehyde	100, 200 and 400 mg/kg of Diet	NE	NE	↑ off-flavour intensity
[130] Bovine	F	Kocetin™-Quercetin	21 and 42 ppm 10% quercetin	-	-	42 ppm ↑ pH of loin.

^a^Feeding phase: *I* initial, *G* growing, *F* final

^b^ *ADG* average daily gain, *BWG* body weight gain, *FE* feed efficiency, *FI* feed intake, *NE* no effect

^c^ *TBARS* thiobarbituric acid reactive substances

Moreover, recent studies have suggested dietary supplementation with ferulic acid, a member of the hydroxycinnamic acid family that can be isolated from agro-industrial by-products [19, 20, 79], as an alternative to the β-adrenergic agonists that are used in the final phase of intensive fattening of beef cattle. Similar to the effect observed with a commercial β-adrenergic agonist treatment, a 12% improvement in feed efficiency and productive parameters has been observed with the use of ferulic acid in steers [20]. Due to the chemical characteristics of ferulic acid, it can be assumed that other phytochemical hydroxycinnamic acid derivatives would exert a similar effect.

## Carcass quality

Carcass quality depends on the intended market for the carcass and its products, but the most important traits in the three species that are considered here are quality grade, yield grade and carcass weight. For these species, the most relevant parameters used to evaluate carcass quality are carcass yield, commercial cuts, breast yield meat (broilers), the ribeye area, fat thickness at the 12th rib, conformation, marbling, cut yield and lean yield (ovine, bovine and porcine) [119, 120].

In terms of these characteristics, the use of antibiotic growth promoters (poultry and swine) and animal metabolism modifiers (pigs and cattle) results in an increase in the size of the breast muscle (broilers), an increase in the ribeye area and a decrease in the deposition of subcutaneous fat (mainly pigs and cattle) [1–3]. However, there are few studies with phytochemicals that assess carcass quality characteristics, and based on the literature reviewed, dietary supplementation of broilers with phytochemicals (Biostrong® 505 and 510, Delacon Biotechnik, Steyregg, Austria; thyme and cinnamon extract, clove powder, agronomy extract and lemon balm, essential oil blend) improved quality parameters, including carcass weight, breast weight, and carcass dressing percentage and reduced abdominal fat and relative abdominal fat [29, 41, 90, 104, 122]. Other studies reported no changes in these parameters [62, 98, 102], and only two studies analyzed the impact of dietary phytochemicals on carcass parameters in pigs. In this sense, Herrera et al. [19] and Li et al. [87] reported a decrease in fat deposition and an increase in pH up to 45 min *postmortem* with the dietary inclusion of ferulic acid. González-Rios et al. [20] reported an improvement in cattle carcass traits and wholesale cut yield with ferulic acid supplementation, which was similar to observations in the positive control group. Similarly, Cardozo et al. [52] noted an improvement (minimal) in the carcass characteristics of cattle supplemented with cinnamaldehyde that was similar to the positive control, and Meyer et al. [117] observed an improvement on carcass dressing after supplementation with a mixture of essential oils and tylosin in steers. In sheep, Chaves et al. [122] and Macias-Cruz et al. [79] observed no effect on carcass characteristics in animals supplemented with cinnamaldehyde and ferulic acid, respectively.

## Meat quality

Generally, meat quality can be defined as the sum of the chemical, physico-chemical, nutritional, sensory, health and food safety characteristics that would yield greater acceptance and a higher price in the market [123–125]. There are several factors that affect meat quality throughout the production chain, from the primary producer to the consumer. One of the animal-related factors is the use of growth-promoting substances in animal production, and several effects on meat quality have been observed, such as decreased fat content to the detriment of the color parameters, decreased tenderness, antimicrobial resistance, and food poisoning, among others [126–129].

However, there are few studies that evaluate the incorporation of phytochemicals into diets to improve meat quality because their use has primarily focused on decreasing the lipid oxidation of meat through direct incorporation. The best results have been found in broilers, which showed improvements in not only productive performance, i.e., carcass quality (carcass yielding), but also meat quality (lower fat content) [104]. In pigs, the most noticeable effects have been observed on the reduction of lipid oxidation [87]. However, the effects in cattle have been inconclusive [130].

### Chemical and physicochemical characteristics

The chemical and physicochemical characteristics of meat largely determine the attributes that are most valued by the consumer, which are its nutritional and sensory aspects [131, 132]; thus, evaluating these parameters is of importance. Hence, Marcinčák et al. [104] observed changes in the composition of the thigh muscle of broilers (higher proportion of crude protein and lower proportion of fat) that were fed with diets containing clove powder, *Agrimonia eupatoria* and *Melissa officinalis* extracts, and Li et al. [87] reported a reduction in shear force values and lipid oxidation of the *M. longissimus dorsi* (LD) in pigs that were given a diet with ferulic acid and vitamin E. Jiang et al. [32] and Kang et al.[130], using soy isoflavone as a phytochemical in broiler feed (40 mg /kg) and quercetin (42 ppm) in cattle, respectively, and observed an increase in water holding capacity (WHC) and pH of the meat. This last parameter, the final pH value in meat, is closely related to the *antemortem* glycolytic potential because a low glycolytic potential has been observed in other studies of antioxidants and has resulted in an increase in pH [133]. In contrast to the results observed by previous authors, Yang et al. [134], Hong et al. [90], Zhou et al. [110], Kang et al. [130], Rossi et al. [112] and Goliomytis et al. [98] reported similar effects between phytochemical treatments and control diets on color parameters, shear force, cooked weight loss, fat content, pH, drip loss, and WHC.

### Nutritional characteristics

For decades, fat content has been the most important nutritional factor to the consumer [132, 135, 136], but at present, the type of fatty acids and cholesterol are also important to the consumer in terms of health. This is due to the relationship between the consumption of saturated fats (SFA) and high serum cholesterol and an increased probability of acquiring diseases such as obesity and high blood pressure, cancer and heart disease [137, 138]. Therefore, the use of synthetic growth promoters is both a production strategy as well as an option to decrease fat deposition and thus satisfy consumer preferences for lean meats [139]. However, in terms of the type of fat, there are few studies that have demonstrated positive changes in the fatty acid profile and cholesterol [140], and moreover, there are few in vivo studies that have evaluated whether the addition of phytochemicals modifies the fatty acid profile. Zhou et al. [110] reported an increase in the concentration of unsaturated fatty acids in the meat of pigs consuming extracts of the herb *Coptis chinensis*, and, in a similar study, Kamboh and Zhu [89] observed that the proportion of total polyunsaturated fatty acids (PUFA), the omega fatty acid ratio (n-6/ n-3) and the PUFA/SFA ratio in broiler breast muscles were significantly improved with the inclusion of different levels of the bioflavonoids genistein and hesperidin. Furthermore, Avila-Ramos et al. [100] observed similar fatty acid composition values in poultry meat treated with oregano essential oils and a control diet. However, no studies in cattle were found for inclusion in this review. One study reported that the addition of cinnamaldehyde decreased the PUFA bio-hydrogenation of the forage being fermented in the rumen [141], indicating that phytochemicals may indirectly carry out their antioxidant activities by protecting this type of fatty acid from bacterial bio-hydrogenation and cause an increase in the accumulation of unsaturated fatty acids in muscle.

### Sensorial characteristics

The organoleptic, or sensory, quality is one of the most important attributes influencing the purchase of and consumer preference for meat. Meat color is one of the sensory criteria considered at the time of purchase [142, 143], and the apparent color depends on the species. The appreciation of meat color can be influenced by several factors, such the degree of fat infiltration in meat (marbling), where high values of intramuscular fat increase light reflectance and therefore create a clearer appearance [144]. However, for decades, the decrease in the intramuscular fat content (mainly pigs, cattle and sheep) using growth-promoting substances has negatively impacted the color, tenderness and juiciness of the meat [142], which are sensory attributes that most influence consumer acceptance. In particular, tenderness plays a decisive role.

Regarding the use of phytochemical feed additives, studies have reported positive effects on several sensory attributes in poultry and swine. Hong et al. [90] found that the meat from broilers supplemented with a blend of essential oils (oregano, anise and citrus peel) was described by the panelists as juicier and more flavorful. In contrast, Goliomytis et al. [97] and Yan et al. [44] found the sensory parameter values in animals (poultry and swine, respectively) supplemented with phytochemicals to be similar to those fed the negative control diet. In ruminants, González-Rios et al. [20] reported better tenderness, juiciness and flavor values for meat from the cattle supplemented with ferulic acid for 30 days, while Chaves et al. [122] reported a higher off-flavor appreciation value in ovine meat supplemented with hesperidin.

### Oxidative stability

One of the most important aspects for the meat industry is to extend the shelf life by delaying or avoiding the oxidation of lipids and / or proteins to preserve quality [38, 145, 146]. Oxidative processes during the shelf life of meat can decrease its sensory and nutritional values, such as color, taste and tenderness [147], and the incorporation of antioxidants in the meat matrix has been a strategy for maintaining meat quality during storage. However, the main challenge for the food industry is currently the replacement of traditional antioxidants (synthetic) with natural antioxidants in the food matrix, and an important factor is the affinity of the antioxidant for the food matrix. Additionally, the methods used to incorporate antioxidants directly into the meat and meat products and the use of packaging that releases active antioxidants are of great importance [148–150]. Currently, one of the most intriguing methods, which was first proposed a few decades ago, is the inclusion of natural antioxidants from animal production [151, 152]. This strategy is of interest to the meat industry because if antioxidants are deposited in the meat during the life of the animal, the addition of exogenous antioxidant compounds would be unnecessary after slaughter, and studies suggest that this method would provide great benefits in terms of animal health and the shelf life of meat by improving the oxidative status *antemortem* [133]. Thus, obtaining an antioxidant with a greater affinity for the matrix would increase the oxidative stability of meat [152, 153]. However, it is noteworthy that, in the case of the ruminant digestive system, the biological activity of phytochemicals is dependent on ruminal pH, which can reduce their antioxidant activity [114]. Major activity occurs at a lower pH, but some compounds can change their structure; oxidation causes a conformational change that reduces their possible effects on the meat matrix [154].

Several studies have concluded that the addition of phytochemicals (broiler: isoflavone, quercetin, naringin and hesperidin; phytochemical additives mixture, oregano essential oil and plant extracts of *Verbenaceae* leaves; bovine: ferulic acid) to animal feed exerts a protective effect against lipid oxidation and maintains low TBARS (thiobarbituric acid reactive substances) values in meat [20, 32, 109, 155, 156]. Another study [87] observed a synergist effect of ferulic acid and vitamin E in pigs that prevented the lipid oxidation of meat.

On the other hand, Bruno et al. [113] reported similar MDA (malondialdehyde) values between tylosin and phytochemical blend treatments. Additionally, a study on effects of dietary oregano essential oil and vitamin E in broilers reported pro-oxidant effects and higher MDA values [100].

## Safety

Although phytochemical feed additives have been perceived as a relatively low risk compared with synthetic growth promoters, even when a product or compound is natural, it is not necessarily safer than other products, and it can equally produce toxicity or other adverse effects [156, 157]. Recently, there has been increased discussion about the safety of herbs used in human medicine, which includes arguments on the inherent adverse effects of some phytochemicals (e.g., capsaicin, the phytoestrogen genistein, and resveratrol) and the lack of safety assessment of most phytochemicals [158–161]. Nevertheless, most of the phytochemicals used in experimental or animal production are still in their early stages (setting, type of phytochemical, dose and exposure period), therefore there is little to no information about the possible negative effects of the phytochemical addition to animal feed on animal and human health [156, 162]. Additionally, little is known about the identification of compounds that are present in the evaluated additives, as most additives are complex extracts that are often mixtures of various plant extracts [13, 15]. Therefore, there are now requirements to identify the chemical components of the evaluated phytochemical feed additives, to ascertain their quality (e.g., metal content), to perform safety tests using several technologies (predictive toxicology of constituents with *in silico* modeling and omics) and to understand the pharmacodynamics and pharmacokinetics as complementary information (absorption, biotransformation, excretion and deposition of these compounds and their derivatives) [160, 161].

## Conclusions

There have been few advances in the use of phytochemicals as growth promoters, and most have been in broilers. Therefore, it is necessary to conduct further research to evaluate changes in the productive performance and the mechanisms of action by which these compounds exert their effects to optimize their use, especially in terms of appropriate doses and exposure periods. Therefore, while their positive effects on growth performance and meat quality have been observed in poultry and pigs, more research is needed to establish safe and effective supplementation programs, doses and productive parameters that clarify the benefits of phytochemical feed additives on the links of the meat production chain, mainly for primary producers. Furthermore, studies are needed to integrate the phytochemical dietary supplementation of animal feed to other production strategies to achieve the best results, and the timely introduction (production phases), phytochemical form (blends or single compounds) and type of phytochemicals that are appropriate for each species should be evaluated.

According to this review, the benefits of phytochemical feed additives will replace the observed effects of traditional growth promoters on quality parameters in meat production in the future. The addition of phytochemicals (plant extracts) during the first production phases, primarily in poultry and pigs, and the use of isolated phytochemicals during the finishing phase will obtain positive effects, mainly in pigs and cattle. Finally, although these additives are considered "natural" products, they should be evaluated for any adverse effects on human and animal health as well as possible interactions with other dietary ingredients.

## Abbreviations

AC: Antioxidant capacity
ADFI: Average daily feed intake
ADG: Average daily gain
AGP: Antibiotic growth promoters
BW: Body weight
BWG: Body weight gain
CFI: Cumulative feed intake
CHOL: Cholesterol
DMI: Dry matter intake
FA: Fatty acid
FAP: Fatty acid profile
FCR: Feed conversion ratio
FI: Feed intake
GSH-Px: Glutathione peroxidase
Hb: Hemoglobin
HCT: Hematocrit
LD: *Longissimus dorsi* muscle
MDA: Malondialdehyde
ND: No difference
NE: No effect
PFA: Polyunsaturated fatty acid
SFA: Saturated fatty acid
SOD: Super oxide dismutase
TBARS: Thiobarbituric acid reactive substance
TGL: Triglycerides
UFA: Unsaturated fatty acid
VLDL: Very low density lipoprotein
WHC: Water holding capacity
